# Northern Indiana Association

**Published:** 1897-05

**Authors:** 


### NORTHERN INDIANA ASSOCIATION.

The twelfth semi-annual meeting of the Northern Indiana and Southern Michigan Homeopathic Medical Association was held in Elkhart, Indiana, Tuesday afternoon, April 27th, in the parlors of the Century Club. Dr. John Borough presided. The members present were: Drs. S. O. Buchtel, Auburn; C. C. Matthews, Leesburg; T. C. Buskirk, White Pigeon; J. C. Huntsinger, Volinia, Mich.; John Borough, Mishawaka; G. L. Shoemaker, Nappanee; W. B. Page, Middlebury; W. H. Thomas, A. L. Fisher, Porter Turner, and H. A. Mumaw, Elkhart.

A number of letters and telegrams from absent members were read. All wished the society a pleasant and profitable meeting and continued prosperity.

Mr. W. J. Ehrhart, representing Boericke & Tafel, homeopathic pharmacists, Chicago; Mrs. Geo. B. Duncan, M. D., Constantine, and Mr. A. S. Zook, Goshen, were among the visiting friends of the Association.

The meeting was called to order at 1.15 o'clock, and, after roll-call, the minutes of the previous meeting were read by the Secretary, Dr. H. A. Mumaw, and approved. The names of Drs. Geo. B. Duncan, Constantine, and H. W. Pierson, editor *Hahnemannian Advocate*, Chicago, were proposed for membership. In the absence of Drs. Lockwood and Kinyon, the Chair appointed Drs. Turner and Buchtel to serve with Dr. Buskirk as a Committee on Credentials. The report was favorable, and the election of candidates unanimous.

Dr. T. P. Wilson, the veteran practitioner, teacher, poet, and humorist, of Detroit, was elected an honorary member.

The President then read his annual address, which was an able exposition of the homeopathic philosophy in particular, and the practice of medicine in general. "Honesty with his patients," said the paper, "as well as with all his other fellow-men, is one of the cardinal points in the physician's experience." The paper was referred to the Committee on Publication, and a vote of thanks extended to the author.

After the collection of annual dues and the disposition of some other miscellaneous business the reports of bureaus was in order. Chairmen: Surgery, Dr. C. S. Fahnestock; Ophthalmology and Otology, Dr. W. B. Kreider; Materia Medica, Dr. C. C. Matthews; Practice, Dr. A. L. Fisher; Gynecology, Dr. G. L. Shoemaker; Pediatrics, Dr. A. A. Leib. The following papers were read and carefully discussed: "The Fundamental Principles of the Law of Cure," by Dr. H. W. Pierson, Chicago; "Treatment of Uraemic Poisoning," by Dr. Chas. Mack, La Porte, read by Dr. Fisher; "How Shall We Prescribe," by Dr. Matthews; "Two Cases of Cholelihiasis," by Dr. C. F. Ellis, Eureka Springs, Arkansas, read by Dr. Fisher; "Lithemia," introductory by Dr. Buchtel; "Neuralgia of the Ovary," by Dr. David Duncan, Professor of Obstetrics, National Medical College, Chicago, read by Dr. Fisher; "Argentum Nitricum," by Dr. Fisher. Reports of cases, by Drs. Matthew, Page, Turner, and Buskirk.

Dr. Buchtel was instructed to complete his paper on "Lithemia," and carefully revise the same for publication in pamphlet form.

Hon. A. S. Zook, who was in attendance by special invitation, delivered an impromptu speech on the "Legal Aspects of Medicine," which was listened to with great interest. The new Indiana medical law was fully explained, malpractice cases cited, and suggestions given on rendering expert testimony.

A hearty vote of thanks were extended Mr. Zook for his instructive remarks, and an invitation to attend future sessions of the Association.

Chairmen of Bureaus for the next meeting are: Surgery,

Dr. Page; Opthalmology and Otology, Dr. W. B. Kreider; Materia Medica, Dr. Buskirk; Practice, Dr. G. B. Duncan; Gynecology, Dr. Matthews; Pediatrics, Dr. Thomas.

The election of officers for the ensuing year resulted as follows: President, Dr. Geo. L. Shoemaker; First Vice-President, T. C. Buskirk; Second Vice-President, Dr. W. B. Page; Secretary and Treasurer, Dr. H. A. Mumaw.

The Chair appointed Drs. Buchtel, Huntsinger, and M. K. Kreider a Committee on Credentials, and Mumaw, Fisher, and Page on Publication. Dr. W. A. Whippy was appointed necrologist.

A vote of thanks was extended to the Century Club for the use of their pleasant and commodious parlors.

It was decided to hold the next meeting in Elkhart on the last Tuesday in September, 1897.—*Elkhart Daily Truth.*

